# Diet and lifestyle habits associated with caries in deciduous teeth among 3- to 5-year-old preschool children in Jiangxi province, China

**DOI:** 10.1186/s12903-018-0686-0

**Published:** 2018-12-20

**Authors:** Liwei Zeng, Yixuan Zeng, Yin Zhou, Jianqiong Wen, Li Wan, Xiaoyan Ou, Xiaojun Zhou

**Affiliations:** 1 Affiliated Stomatological Hospital, Nanchang University & Jiangxi Province Key Laboratory of Oral Biology Medicine, Nanchang, 330006 Jiangxi China; 2 School of Public Health, Nanchang University & Jiangxi Province Key Laboratory of Preventive Medicine, Nanchang, 330006 Jiangxi China

**Keywords:** Children, Dental caries, Caries prevalence

## Abstract

**Background:**

The purpose of this study was to investigate the status of caries in deciduous teeth and the effect of diet and lifestyle habits on dental caries among 3- to 5-year-old preschool children in Jiangxi Province, China.

**Methods:**

In total, 2880 cases involving preschool children were selected by stratified cluster sampling. The dental examination methods and criteria followed the WHO guidelines. SPSS 19.0 was used for the statistical analysis. Chi square tests were used to compare the caries prevalence among children with different social characteristics. Non-parametric tests were used to compare the decayed, missing and filled teeth (dmft) index values. Univariate and multivariate regression analyses were used to study the effect of diet and lifestyle habits on dental caries.

**Results:**

The caries prevalence among the 2880 cases of 3- to 5-year-old preschool children in Jiangxi Province, China was 49.13%. There was no gender difference in this rate (*P* > 0.05). The caries prevalence increased with age (*P* < 0.05). The prevalence of caries in the rural areas was higher than that in the urban areas (*P* < 0.05). The deciduous central incisors and deciduous molars had a higher caries prevalence than the other deciduous teeth. According to the multivariate logistic regression analysis, the caries risk increased with living in a rural area, exclusive breastfeeding, greater frequency of daily snacking, high frequency of snacking before sleep and beginning to brush teeth at a late age; the caries risk decreased when parents helped their children brush their teeth.

**Conclusion:**

The caries prevalence among 3- to 5-year-old preschool children in Jiangxi was lower than the level throughout the country and lower than the rate in other developing countries. The children’s diet and lifestyle habits were closely related to dental caries. Parents and children should be more aware of oral health, and parents should help their children develop healthy lifestyle behaviours.

## Introduction

Caries is a common disease and is frequently encountered among preschool children in many countries [1, 2]. The rapid development and extent of tooth surface damage as characteristics of childhood caries have attracted great attention in clinical treatment. Dental caries is a progressive disease with no self-healing properties; thus, if tooth decay is not treated in a timely manner, deciduous tooth caries may cause apical periodontitis and pulpitis, and permanent tooth germs may be infected, affecting the development of permanent teeth or causing the premature loss of deciduous teeth, ultimately resulting in the crowding of permanent teeth [3] or increasing the risk of subsequent caries in the permanent dentition [4]. Children who suffer from severe caries will resist chewing because of pain, which affects the development of facial movement and language ability and even causes trouble sleeping [5–7]. Currently, although advanced clinical treatment and filling materials are used to treat coresidential-pain problems, chewing difficulty caused by caries [8, 9] still affects the growth and quality of life of preschool children for a limited period [10–12]; therefore, starting from the source and preventing the production of dental caries is a better choice than caries treatment. The existing literature suggests that the appearance and development of childhood caries is related to many factors, such as feeding patterns [13], the frequency of sugary food intake [14, 15], brushing habits [16], parents’ education level [17] and the family’s economic situation [17–19]. Among the above factors, the parents’ education level and the family’s economic situation in the context of dental caries formation are difficult to change, but children’s oral health-related behaviours and lifestyle can be easily adjusted and managed by the caregiver, which inspires us to implement more behavioural interventions. It is regrettable that there are few reports about dental health related to preschool children in the Jiangxi province of China. Therefore, this study can fill in gaps in these data and provide comparative data for similar future research. It also provides support for government departments to improve children’s oral health and formulate specific regional strategies for the prevention and control of dental caries.

## Materials and methods

### Participants

This study focuses on 3- to 5-year-old preschool children in the Jiangxi province of China. The aim was to explore the association between the children’s deciduous teeth caries and their daily lifestyle factors. Jiangxi province is a typical highland terrain located in the south of China, containing 11 cities and 100 counties. It is an agricultural province in which the major crop is rice. Jiangxi is a less-developed inland province. Most of the cultivated land is barren loess land. The population of Jiangxi is more than 95% Han people. Consequently, the deciduous teeth caries prevalence discussed here may be significantly different from those of other areas.

### Sample size and sampling method

The number of deciduous teeth caries was obtained from the report of the Third National Oral Health Survey in 2005, and the caries prevalence was *P* = 66% for 5-year-old children [20]. The permissible relative error of the total *P* value was controlled within 10%, and *deff* = 4 for this sampling design. Therefore, the sample size was 792 for 5-year-old children. The sampling method was the same as above for both 3- and 4-year-old children. The total sample size for these three age groups was at least 792 × 3 = 2376. In this study, the actual sample size of the 3-year-old group was 952; the size of the 4-year-old group was 978; the size of the 5-year-old group was 950; and the total sample size was 2880. The sample size achieved the minimum requirement for the sampling method, which guaranteed the validity of the results. A stratified cluster sampling method was applied in this study. (1) According to the geographical distribution of cities in Jiangxi province, five city regions were chosen. Considering the capital city Nanchang as the central region, four other cities were selected as follows: an eastern region (Shangrao); a southern region (Ganzhou); a western region (Yichun); and a northern region (Jiujiang). (2) In each of the above cities, one urban district and one rural county were chosen using a simple random sampling method. Therefore, 10 districts/counties were selected in total. (3) In each of the above districts/counties, three sub-districts/towns were picked using the above method. (4) One kindergarten was singled out in each of the 30 sub-districts/towns. An equal distribution was applied to each location and age group. Therefore, approximately 96 children were selected from each kindergarten.

### Clinical examination of dental caries

Clinical examinations of the children were conducted by three dentists with at least 10 years of oral clinical working experience. Unified training sessions were provided to clinical examiners from all the provinces, before the national survey began. The training course included inspection procedures, disease diagnosis and standard scoring. Each dentist accepted theoretical and clinical training and every dentist and a reference dentist carried out the examination on 10 to 15 pre-subjects to assess the consistency. After training, the clinical examination results of all three dentists passed the standard consistency test. The Kappa values for the three dentists were 0.97, 0.97, and 0.99.

Clinical dental caries were diagnosed according to WHO criteria. Dental clinical examinations were carried out with participants seated on a chair, using artificial light, plane mouth mirrors and standard WHO CPI probes [21]. A cotton ball was used to clean off material on the tooth surface. The dental health condition of each tooth was recorded in detail. Re-examination was conducted for a random 5% of samples every day. Decayed, missing and filled teeth (dmft) index values and caries prevalence were used to estimate the caries status.

### Questionnaire survey

Questionnaire surveys were completed by the parents of the children participating in this study. The questionnaire included (1) demographic characteristics (the children’s age and gender and the parents’ education level, nationality, and residence); (2) daily diet and lifestyle factors related to the children’s oral health, such as the frequency of sugary food intake, age at starting to brush, tooth brushing frequency, fluoride toothpaste use, parent-assisted brushing; (3) and the oral health knowledge of the parents. The evaluation included a series of judgement questions about oral health knowledge (two investigators trained on the questionnaire were present at the scene to provide 1-on-1 help to the parents as they completed the questionnaires).

### Data entry and statistical analysis

Epidata 3.0 was used to enter the data. The dental caries prevalence and dmft index were analysed using SPSS 19.0 statistical software. The dmft index is represented as the mean ± SD. Chi square tests were used to compare the significant differences in caries prevalence among children of different genders, ages and residences; a non-parametric test was used to compare significant differences in the dmft index among children of different genders, ages and residences. Univariate logistic regression was used to study the effect of single independent variables on dental caries; multivariate logistic regression was used to study the effect of multivariate interactions on dental caries. Whether or not a child suffered from caries was a dependent variable in logistic regression, and the odds ratio (OR) was used to indicate the degree of association between variables and caries. The statistical significance level for all tests was 0.05.

## Results

### Caries status of 3- to 5-year-old children

In the 2880 3- to 5-year-old children, 1415 had caries with 6697 carious teeth in total. The caries prevalence was 49.13% and the dmft index was 2.33 ± 3.549. For the carious teeth, the caries decay rate was 98.97%. The caries filling prevalence was 1.00%, and the caries loss prevalence was 0.03%. The caries prevalence was 48.86% (727/1488) for boys and 49.43% (688/1392) for girls. There was no gender difference (*χ*^*2*^ *=* 0.093*, P* > 0.05). The caries prevalence increased as age increased, and the increase showed statistical significance (*χ*^*2*^ *=* 97.132*, P <* 0.001). The residence factor also appeared to be statistically significant. The caries prevalence was higher in rural children than in urban children (*χ*^*2*^ *=* 51.866*, P <* 0.001), as shown in Table 1.

**Table 1:** Caries status among 3- to 5-year-old children of different genders, ages and residences

Variables		Number of participants	Caries status			Dmft		
			Prevalence (%)	*χ* ^*2*^	*P*	Mean ± SD	*Z/H* ^a^	*P*
Gender	Boys	1488	48.86	0.093	0.395	2.30 ± 3.497	−0.305	0.760
	Girls	1392	49.43			2.36 ± 3.605		
Age	3	952	37.29	97.132	0.000	1.42 ± 2.672	122.955	0.000
	4	978	50.31			2.41 ± 3.557		
	5	950	59.79			3.15 ± 4.064		
Residence	Urban	1428	42.37	51.866	0.000	1.79 ± 3.032	−8.156	0.000
	Rural	1452	55.79			2.86 ± 3.922		
Total	–	2880	49.13	–	–	2.33 ± 3.549	–	–

^a^Data are non-normally distributed; non-parametric tests, including the Wilcoxon rank sum test and Kruskal-Wallis *H* test, were used

**Fig. 1:**
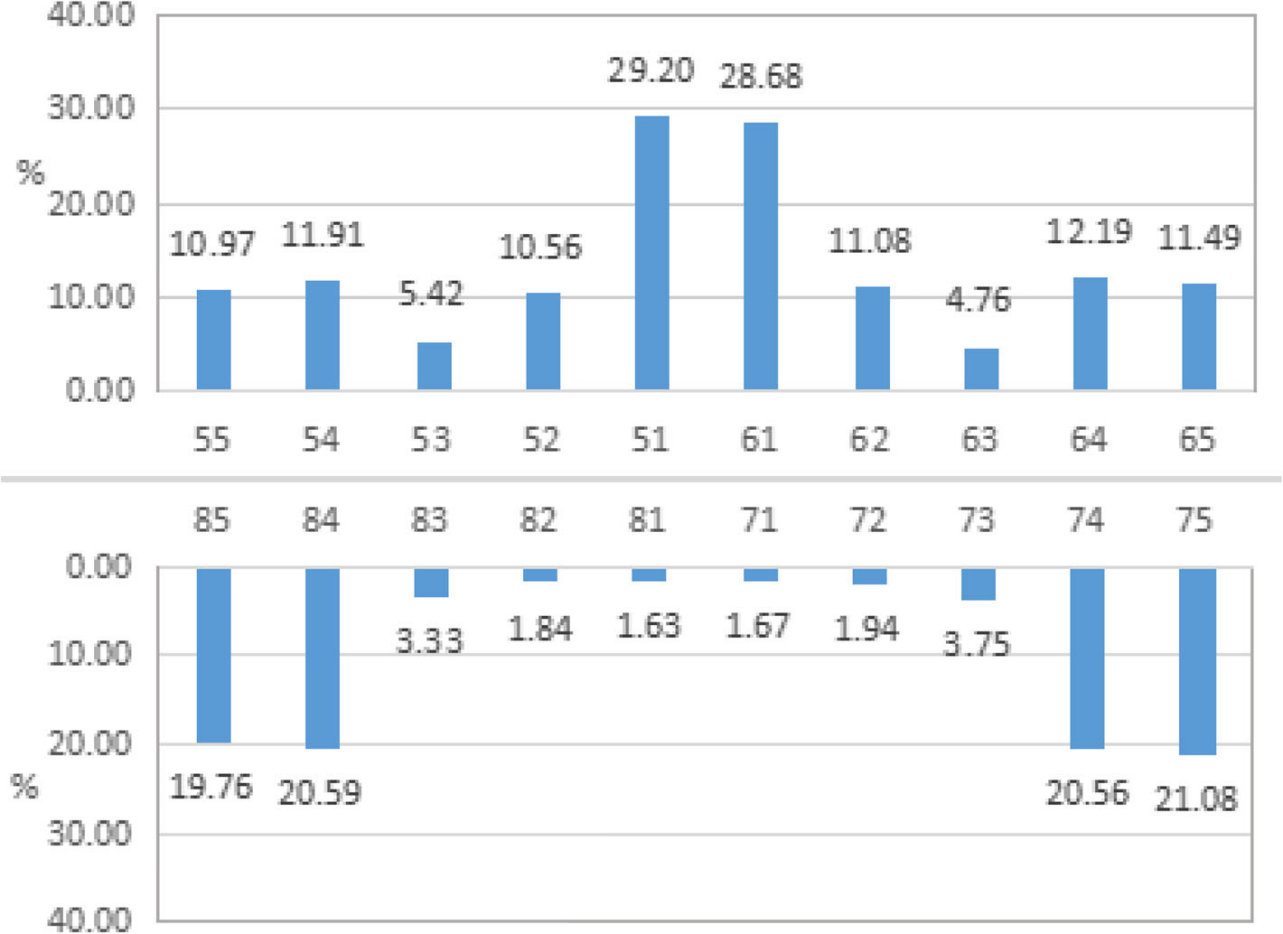
Caries prevalence in different tooth positions

The caries location distribution is presented in Fig. 1. The data indicate that the maxillary central incisors had the highest caries rate at 29.20%. The caries prevalence was 21.08% for mandibular second molars and 20.59% for mandibular first molars. The caries risk was higher for maxillary teeth than for mandibular teeth (*χ*^*2*^ *=* 17.735*, P <* 0.001). The caries prevalence also showed a significant difference for different tooth positions (*χ*^*2*^ *=* 708.691*, P <* 0.001).

### Effects of diet and lifestyle factors on caries in children

The 2880 children’s caries-related factors were analysed by univariate regression analysis, which showed that the prevalence of caries in exclusively breastfed children was the highest at 54.25% and that the risk of caries was higher for breastfed children than for formula fed children (*P* < 0.05). The rates of children who had sugary snacks, sugary drinks, and sugary milk/yogurt at least once a week were 61.14, 26.05, and 65.35%, respectively. There was a significant difference between the children who suffered from caries and those who did not in the intake frequency of sugary snacks and sugary drinks (*P* < 0.05), but there was no significant difference between the children who suffered from caries and those who did not in the frequency of sugary milk/yogurt intake (*P >* 0.05). Children who often snacked before sleep had a higher risk of caries than those who never snacked before sleep, and the difference was statistically significant (*P* < 0.001). The univariate regression analysis of the home environment and brushing habits of the children showed that the rural children had a higher risk of caries; their risk was 1.262 times higher than that of the urban children. Approximately 59.06% of the children began brushing their teeth before they were 3 years old, and our analysis showed that the prevalence of caries for children who began to brush their teeth at the age of 4 was higher than the prevalence for children who started brushing their teeth before the age of 1 (*P* < 0.001). Children who brushed their teeth once a day had the highest caries prevalence (52.18%), but this difference was not significant compared with other brushing frequencies (*P* > 0.05). In addition, univariate analysis also showed that the use of fluoride toothpaste and parent-assisted brushing were two factors that were not significantly associated with caries (*P* > 0.05), as shown in Table 2.

**Table 2:** Univariate and multivariate logistic regression analysis of factors associated with caries

Variables	Categories	Frequency (%)	dmft (_Mean ± SD_)	Prevalence (%)	Univariate analysis^a^		Multivariate analysis^b^	
					OR	95%CI	OR	95%CI
Diet habits								
Breastfeeding	Exclusively breastfed	36.35	2.71 ± 3.808	54.25	1		1	
	Predominantly breastfed	17.05	2.27 ± 3.524	48.68	0.948	0.795–1.132	0.770	0.616–0.961*
	Mixed feeding (50/50)	17.12	1.82 ± 3.016	44.02	0.786	0.658–0.939**	0.676	0.542–0.844***
	Exclusively formula-fed	20.14	2.08 ± 3.409	45.52	0.835	0.709–0.984*	0.687	0.557–0.847***
	Predominantly formula-fed	9.34	2.40 ± 3.608	47.21	0.894	0.704–1.136	0.746	0.566–0.983*
Frequency of sugary snack intake	Seldom / never	38.85	1.95 ± 3.287	44.68	1		1	
	One time a week	12.95	1.89 ± 3.271	45.58	0.837	0.683–1.027	1.072	0.839–1.371
	2–6 times a week	20.17	2.39 ± 3.499	51.46	1.060	0.901–1.248	1.222	0.987–1.513
	One time a day	17.53	2.83 ± 3.888	54.06	1.177	0.988–1.402	1.320	1.051–1.657*
	≥2 times a day	10.49	3.27 ± 4.017	57.28	1.341	1.068–1.685*	1.411	1.057–1.883*
Frequency of sugary drink intake	Seldom / never	73.96	2.18 ± 3.415	48.22	1		1	
	One time a week	9.31	2.53 ± 3.988	45.90	0.848	0.667–1.079	0.876	0.669–1.147
	2–6 times a week	7.92	3.03 ± 3.951	57.89	1.375	1.057–1.788*	1.232	0.917–1.655
	One time a day	5.73	2.66 ± 3.741	52.73	1.115	0.822–1.514	0.955	0.679–1.343
	≥2 times a day	3.09	2.80 ± 3.590	51.69	1.070	0.706–1.621	0.834	0.518–1.342
Frequency of sugary milk/yogurt intake	Seldom / never	34.65	2.32 ± 3.497	48.70	1		1	
	One time a week	7.40	1.98 ± 3.388	44.13	0.790	0.603–1.035	0.803	0.589–1.096
	2–6 times a week	15.56	2.48 ± 3.727	50.45	1.018	0.846–1.225	0.930	0.736–1.175
	One time a day	30.97	2.21 ± 3.423	49.22	0.969	0.850–1.105	0.911	0.753–1.103
	≥2 times a day	11.42	2.67 ± 3.864	51.67	1.069	0.861–1.327	0.942	0.721–1.230
Frequency of snacking before sleep	Never	39.34	1.94 ± 3.173	45.01	1		1	
	Occasionally	50.31	2.39 ± 3.603	50.10	1.004	0.906–1.113	1.213	1.027–1.434*
	Often	10.35	3.46 ± 4.309	60.07	1.504	1.193–1.897***	1.730	1.315–2.277***
Lifestyle habits								
Residence	Urban	49.58	1.79 ± 3.032	42.37	1		1	
	Rural	50.42	2.86 ± 3.922	55.79	1.262	1.138–1.399***	1.653	1.418–1.928***
Age at starting to brush	Before or within first year	5.38	1.82 ± 2.957	42.58	1		1	
	2nd year	21.84	1.96 ± 3.274	44.99	0.818	0.699–0.957*	0.895	0.629–1.273
	3rd year	31.84	2.64 ± 3.777	52.56	1.108	0.973–1.261	1.155	0.818–1.632
	4th year	11.18	3.07 ± 3.904	61.49	1.597	1.276–1.999***	1.613	1.084–2.399*
	5th year	2.12	2.98 ± 4.002	47.54	0.906	0.548–1.498	0.923	0.502–1.696
	Never/not sure	27.64	2.00 ± 3.320	44.85	0.813	0.707–0.935**	0.752	0.534–1.058
Tooth brushing frequency	Occasionally or never	27.01	1.95 ± 3.251	44.34	1		1	
	Often, but not every day	2.12	1.64 ± 2.881	42.62	0.743	0.447–1.234	0.606	0.307–1.200
	One time a day	49.38	2.48 ± 3.619	52.18	1.091	0.983–1.211	1.009	0.661–1.540
	≥2 times a day	21.49	2.52 ± 3.763	48.79	0.953	0.814–1.115	1.008	0.665–1.527
Fluoride toothpaste use	Yes	10.38	2.45 ± 3.490	52.51	1		1	
	No	89.62	2.31 ± 3.556	48.74	0.951	0.880–1.027	0.803	0.626–1.031
Parent-assisted brushing	Never	58.19	2.61 ± 3.598	49.76	1		1	
	Occasionally	32.53	2.47 ± 3.779	48.77	0.952	0.838–1.082	0.837	0.685–1.023
	Weekly	0.66	2.16 ± 3.060	57.89	1.375	0.553–3.418	1.368	0.529–3.538
	Daily	8.61	1.96 ± 3.287	45.56	0.837	0.652–1.075	0.739	0.549–0.994*

^a^Univariate logistic regression was used to study the association between single independent variables and caries

^b^Multivariate logistic regression analysis was used to consider the association between all variables and caries

*P < 0.05

**P < 0.01

***P < 0.001

The results of the multivariate logistic regression analysis showed that when considering all variables, exclusive breastfeeding, eating more sugary snacks daily, more frequent snacking before sleep, living in a rural residence, starting tooth brushing at a later age, and having parents who do not help children brush their teeth all increased the risk of caries. Moreover, compared with the results of the univariate regression analysis, the association between the frequency of sugary snack intake and caries was enhanced (*P* < 0.05). The caries risk associated with snacking before sleep occasionally or often was 1.213 times and 1.730 times higher, respectively, than that for never snacking before sleep, indicating that this variable had a greater contribution to caries formation. The risk of caries among the children who began brushing their teeth in their 4th year was 1.613 times higher than the risk among the children who began brushing in their first year, and the association with caries was weakened compared to that revealed by the univariate regression analysis (*P* < 0.05) as shown in Table 2.

## Discussion

The oral health survey in Jiangxi province, which included a thorough review by the Ethics Committee of the Chinese Stomatological Association, is one of the most comprehensive oral health surveys in recent years. Good organizational coordination between relevant governmental organizations and kindergartens in the early stage guarantee the integrity of the investigation and validity of the database. To ensure the scientific nature of the investigation and data accuracy, the field personnel were professionally trained, and Professor Xiaoyan Ou supervised and managed the survey as the technical leader.

In this study, we found that the dental caries prevalence of preschool children aged 3 to 5 years was 49.13% in Jiangxi province, which is higher than the prevalence in Thessaloniki, Greece, (20.20%) and in Bangalore, India (40%) [22, 23]. However, the prevalence is lower than that in Khartoum, Sudan (52.4%) [24], which is a developing country, and it is also lower than that of Riyadh, Saudi Arabia (69.0%) [25]. The prevalence of caries for 5-year-old children in this study was 59.79%, which is lower than the prevalence (70.90%) reported by the National Health and Family Planning Commission of China [26]. There was no gender difference for the caries prevalence between 3- to 5-year-old children in Jiangxi province, and the prevalence increased with increasing age (*P* < 0.05). This finding is slightly different from the results in Sri Lanka [27] and Riyadh [25], where there was no significant difference between gender or age for the prevalence of caries. Such a difference may be due to different statistical analysis methods and sample sizes. Therefore, caries prevention in children should be conducted from an early age. More attention should be paid to the influencing factors of children’s oral health. The prevalence of caries in 3- to 5-year-old children in rural areas was higher than the prevalence in children in urban areas (*P* < 0.05) of Jiangxi province. The caries prevalence in rural families was 55.79%, which was higher than the prevalence in children in a rural area of India (45%) [28].This result indicates that oral health education is insufficient for rural families in Jiangxi province. More information and education are needed to help rural families realize the importance of children’s caries. Considering the position of deciduous tooth dental caries, maxillary deciduous central incisors and mandibular deciduous molars have the highest risk of caries. This finding coincides with the conclusion from A. Cortes’s research on children’s dental caries in Columbia [29]. He also emphasized the influence of radiographic assessment on the incidence of caries. This result means that radiographic assessment needs to be applied in time to evaluate the severity of a decayed tooth when the caries appears, allowing medical treatment can be received as early as possible. The high prevalence of caries in maxillary deciduous central incisors and deciduous molars may be because maxillary deciduous central incisors erupt at an earlier time and due to the poor self-cleaning ability of incisors. Therefore, food residues tend to accumulate on the tooth surface. However, salivary glands are located near the lingual side of the mandibular central incisors. Saliva has a strong ability to flush food residue away. Food usually is chewed and ground up by the molars after it is cut off, and then it is swallowed. Thus, food scraps can easily adhere to and accumulate on the molars. The fossae, grooves and pits on the surface of deciduous molars are more numerous and deeper than those of other deciduous teeth. It is not easy to thoroughly clean molars during tooth brushing; thus, bacteria can decompose the residue to produce acids [30]. It is suggested that children have a proper oral cleaning after their deciduous teeth sprout and rinse their mouth with water after eating to reduce or remove food debris.

Through multivariate regression analysis, we found that exclusive breastfeeding leads to a higher risk of caries compared with that from other forms of feeding. To date, many articles have studied the relationship between feeding methods and dental caries; a study conducted in Sri Lanka [13] found that children who were exclusively breastfed had a higher incidence of dental caries than those who were not exclusively breastfed, which is consistent with the results of this study. In addition, some studies have found that children receiving prolonged breastfeeding for 2 years or more are at a higher risk of early severe caries (S-ECC) [31]. A. Sayegh’s study also showed that breastfeeding over 18 months can cause severe caries (OR = 2.3, 95%CI 1.1–4.8) and gingivitis (OR = 2.9, 95%CI 1.5–5.7) [32]. However, American and Japanese paediatric dentistry organizations recommend that breastfeeding mothers need to be careful with their infants’ oral hygiene after 6 months of age [33]. Because our study investigated the feeding pattern of children within 6 months of birth and the timing and habits of breastfeeding among these children are unknown, further research should focus on this aspect. Breastfeeding is considered to be an essential source of nutrition for infants in the early stages of growth and enhances immunity. WHO recommends exclusive breastfeeding up to 6 months and continued breastfeeding up to 2 years and beyond [34]. Therefore, we believe that breastfeeding is necessary in the process of child development. However, the mouth should be cleaned after breastfeeding; otherwise, if milk is stuck on the tooth surface for a long time, it will produce fermentable carbonic acid compounds, thus increasing the risk of tooth decay.

A more frequent snack intake resulted in higher caries prevalence (*P* < 0.05), and children who often snacked before sleep had higher caries prevalence (*P* < 0.001). These findings regarding carbohydrate-based snack intake were similar to the results reported in D. Declerck’s study, which indicated that drinking sugar-containing drinks at night and sugar-containing drinks between the main meals was significantly associated with caries [35]. A. Xavier indicated that sugar plays a necessary role in the formation of dental caries and that reducing sugar frequency is more important than controlling the amount of sugar intake [36], which revealed that children can prevent dental caries by reducing the frequency of sugar intake. The univariate analysis model revealed that the frequency of sugary drink intake was significantly associated with caries prevalence but that the association was weakened after adjusting for the intake frequency, suggesting that this variable contributes less to caries than the feeding pattern, sugary food intake before sleep and parent-assisted brushing among 3- to 5-year-old children. Regarding the effect of sugary food intake on children, Hery Mwakayoka [37] gave a cautious explanation that the intake of sugary foods at 3 to 4 years old revealed no association with caries because the effects of consuming sugary snacks at 3 to 4 years old would manifest in later years of life as the development of dental caries takes time. Therefore, our study agrees that the impact of sugary food intake on caries in children is a long-term process. A high frequency of sugary food intake for a long period of time can increase the risk of caries, suggesting that parents can prevent dental caries by reducing the frequency of their children’s sugary food intake.

Based on the results of the univariate and multivariate analyses, the children’s residence was related to caries prevalence, suggesting that we should pay special attention to the oral health protection of children in disadvantaged living environments and provide some appropriately directed policies [38]. Starting to brush at an earlier age and parent-assisted brushing can often reduce the risk of caries, which is consistent with the results of other studies [35, 39, 40]. We should encourage parents to help or urge their children to brush their teeth after their first deciduous tooth emerges. Parents, who are the closest caregivers to their children, have great power to influence their children’s behaviour in life [41, 42]. One study showed that the lack of sharing oral health knowledge with their children by parents was a factor associated with caries [5, 43]. Therefore, we encourage parents to provide oral health guidance to their children as early as possible.

A study of Dai preschool children in Yunnan province, China, showed that participants who brushed their teeth every day had a 76% lower risk of caries than those who did not brush their teeth every day (*P* = 0.002) [44]; however, this study did not analyse the association between brushing frequency and dental caries. Dentists recommend brushing your teeth at least twice a day; however, our study showed no significant association between brushing frequency and caries, although 70.87% of the children brushed their teeth 1 or 2 times a day or more. It may be that children cannot effectively remove plaque when they brush their teeth because they are unable to brush their teeth properly in the absence of parental help. In addition, our results show that brushing with fluoride toothpaste does not reduce the risk of caries, but some studies have shown that using fluoride toothpaste can prevent dental caries effectively [45–47]. This discrepancy may be because the content of toothpaste used in this study was not detailed enough and because research on the frequency and duration of fluoride toothpaste use in children is lacking. Therefore, parents should help children develop the habit of brushing their teeth themselves in their early years and help them master the correct method of brushing; parents should also teach their children to clean residue from their teeth consciously and promptly. At the same time, we should consider using fluoride toothpaste in order to enhance the ability to resist caries formation [46, 48].

Comparing the results between univariate and multivariate analysis of the aforementioned variables, feeding methods, sugary snack intake before sleep and parent-assisted brushing are three studied factors that may influence caries. The results indicate that the above three factors have a stronger association with caries in a multivariate model than that in a univariate model, which means that these factors have a strong effect on caries in children.

## Conclusion

The caries prevalence of 3- to 5-year-old preschool children in Jiangxi is lower than the level throughout the country, and it is lower than the prevalence in other developing countries. There was no significant association between gender and the prevalence of caries. Caries prevalence increases as age increases. The prevalence in the rural areas was higher than that in the urban areas. Deciduous central incisors and deciduous molars have a higher caries prevalence than other deciduous teeth. According to multivariate logistic regression analysis, the caries risk increases for children living in rural areas, children who receive exclusive breastfeeding, have a higher frequency of daily snacking, a high frequency of snack intake before sleep and who begin brushing their teeth at a late age; the caries risk decreases when parents help children brush their teeth frequently. Parents and children should have stronger awareness of oral health, and parents should help their children develop healthy lifestyle behaviours.

## Abbreviation

WHO: World Health Organization
